# Effect of body mass index on N-terminal pro-brain natriuretic peptide values in patients with heart failure

**DOI:** 10.1186/s43044-023-00401-1

**Published:** 2023-08-29

**Authors:** Tuan Ha Manh, Duong Do Anh, Tung Le Viet

**Affiliations:** 1 University of Medicine and Pharmacy at Ho Chi Minh City, 215 Hong Bang Str., District 5, Ward 11, Ho Chi Minh City, 700000 Vietnam; 2 Laboratory Department, Sai Gon - Long Khanh Clinic, 57 Nguyen Thi Minh Khai Str., Quarter 5, Ward Xuan An, Long Khanh City, Dong Nai Province 76000 Vietnam; 3 University Medical Center Ho Chi Minh City, 201 Nguyen Chi Thanh Str., District 5, Ward 12, Ho Chi Minh City, 700000 Vietnam

**Keywords:** Body mass index (BMI), Heart failure, Obesity, N-terminal pro-B-type natriuretic peptide (NT-proBNP)

## Abstract

**Background:**

N-terminal pro-B-type natriuretic peptide (NT-proBNP) is a biomarker used for the diagnosis of heart failure. There is a relationship between NT-proBNP levels and body mass index (BMI). The study aimed to explore the impact of BMI on NT-proBNP concentrations and to examine whether other factors independent of or combined with BMI affect NT-proBNP values in patients with heart failure.

**Results:**

A total of 293 participants were recruited. The mean age was 68.9 ± 13.2 years, males accounted for 46.4% of the total cohort, the mean BMI was 23.1 ± 4.0 kg/m^2^, and the median NT-proBNP level was 3776 (1672–8806) pg/ml. There was an inverse relationship between BMI and log NT-proBNP (*r* = − 0.29; *p* < 0.001, Spearman correlation). Each standard deviation increase in BMI (4 kg/m^2^) was associated with a 7% decrease in NT-proBNP values in the total cohort. The independent inverse determinants of NT-proBNP other than BMI were male gender and eGFR, while the variables directly correlated to NT-proBNP were LVEF ≤ 40% and NYHA class III–IV heart failure.

**Conclusions:**

There is an inverse association between BMI and NT-proBNP levels. However, the correlation is weak, and there are other variables that have a significant impact on the NT-proBNP values as well. The NT-proBNP levels are still valuable in the diagnosis of heart failure regardless of BMI status.

## Background

N-terminal pro-B-type natriuretic peptide (NT-proBNP) is a type of natriuretic peptide produced by the ventricular myocardium primarily in response to myocardial stress due to pressure overload or volume expansion [1–5]. NT-proBNP is a biologically inactive component derived from the prohormone of BNP and is secreted into the circulation, so it is also a measurable biomarker used to reflect myocardial stress along with BNP [1]. NT-proBNP is used for diagnosis, follow-up, and prognosis in heart failure [1–3, 6–9]. The NT-proBNP value has been established for the diagnosis of heart failure according to the European Society of Cardiology (ESC) guidelines 2021[6]; however, many studies have shown that NT-proBNP levels could be confounded by many patient-related factors, which results in inaccuracy in the diagnosis of heart failure [2, 3, 7, 10]. Obesity, which is one of the disorders commonly seen in patients with heart failure, affects the NT-proBNP values in the diagnosis of heart failure [2, 3, 10–17] so significantly that many studies have suggested that the NT-proBNP level should be adjusted for the patient’s body mass index (BMI) to help diagnose heart failure more accurately [15, 18, 19]. The association between BMI and NT-proBNP has been studied extensively. However, the issues what extent BMI affects the NT-proBNP levels in patients with heart failure, and whether other factors, including age, gender, or other disorders commonly encountered in heart failure patients, such as diabetes and hypertension play any role in the relationship between BMI and NT-proBNP levels have not yet been examined intensively.

This study aimed to explore the impact of BMI on NT-proBNP concentrations in heart failure patients and to examine whether other factors independent of or combined with BMI affect NT-proBNP values in patients with heart failure.

## Methods

### Study design

This was a descriptive cross-sectional study. All data from the participants were measured and collected upon admission to the hospital.

### Study participants

The participants who were included in the study were more than 18 years of age with New York Heart Association (NYHA) class II-IV heart failure and were admitted to a hospital between May 2021 and May 2022. All participants consented to take part in the study. The participants with renal failure of eGFR < 30 ml/min/1.73 m^2^, malignancy, use of immunosuppressive drugs for more than 14 days, or clinical and laboratory manifestations of severe liver failure were excluded. Sampling was performed in a convenient way. There were 332 participants screened, and 39 patients were excluded due to severe renal failure [10], severe liver failure [4], corticosteroid use [15], or a lack of NT-proBNP measurement [10]. Finally, 293 eligible patients were included in the study.

### BMI categories

Weight and height were measured at admission, and BMI was calculated as weight in kilograms divided by height in metre squared. The patients were classified into three BMI categories based on the WHO’s BMI criteria for Asians: lean (BMI < 23 kg/m^2^), overweight (23 ≤ BMI < 25 kg/m^2^), and obese (BMI ≥ 25 kg/m^2^) [20].

### Data collection

The participants’ data on age, gender, comorbidities (diabetes, hypertension, and kidney diseases), NYHA class of heart failure, BMI, left ventricular ejection fraction (LVEF) index, NT-proBNP levels, and biochemical indices including blood glucose, HbA1c, lipids, creatinine, and glomerular filtration rate (eGFR) were collected to complete the data form at admission. Confounding variables that may affect the NT-ProBNP values [2, 10, 21] were controlled by exclusion criteria, and other confounding variables would be assessed by statistical analysis during data processing. The NYHA class of heart failure and LVEF index were assessed by cardiologists to control for measurement errors.

### Measurement of NT-proBNP

Venous blood was collected into an EDTA anticoagulation tube and processed immediately after sampling. NT-proBNP was measured by electrochemiluminescence immunoassay using a Cobas e602 analyser with Elecsys proBNP II stat reagent (Roche diagnostics, Mannheim, Germany). The assay has a measuring range of NT-proBNP values from 10 to 35,000 pg/ml.

### Statistical analysis

Continuous variables are presented as the mean ± SD (standard deviation) if normally distributed and as the median (interquartile range, IQR) if not normally distributed. Categorical variables are presented as percentages (%). Differences in the baseline characteristics between the groups were analysed using independent samples t test, analysis of variance, Chi-square, Kruskal‒Wallis, and Mann‒Whitney tests, as appropriate. The NT-proBNP values were log-transformed because of their skewed distribution, and the log-transformed NT-proBNP values were used in a multivariable linear regression model to examine the correlation between NT-proBNP values ​​and other variables. The correlation between BMI, other factors, and log NT-proBNP values was determined by Spearman correlation. The impact of BMI on NT-proBNP values was estimated by exponentiating the *β* coefficient determined with multivariable linear regression analysis between the log-transformed NT-proBNP values and covariates. A two-sided *p* value < 0.05 was considered to be statistically significant. Data processing and analysis were performed using STATA version 15.1 software.

## Results

### Baseline characteristics of the study population

Table 1 shows the clinical characteristics and laboratory results of the study patients. The mean age was 68.9 ± 13.2, 46.4% of the patients were male, the mean BMI was 23.1 ± 4.0 kg/m^2^, the mean EF was 40.9 ± 16.2%, patients with NYHA class III–IV heart failure accounted for 63.1%, and the median NT-proBNP value was 3776 (1672–8806) pg/ml. There were no significant differences in gender, EF index, NYHA class heart failure, and comorbidities such as diabetes and hypertension across all BMI categories. Similarly, there were no significant differences in biochemical indices such as HbA1c, eGFR, cholesterol, HDL (high-density lipoprotein) cholesterol, and LDL (low-density lipoprotein) cholesterol levels across all BMI strata, except for triglyceride levels (*p* = 0.043).

**Table 1:** Baseline characteristics of the study population

	Total (*n* = 293)	Lean (*n* = 138)	Overweight (*n* = 72)	Obese (*n* = 83)	*p* value
Age (years) *(mean ± SD)*	68.9 ± 13.2	72.0 ± 12.8	68.7 ± 10.9	63.8 ± 14.3	< 0.001^b^
< 50	21 (7.2)	5 (3.6)	1 (1.4)	15 (18.1)	< 0.001^a^
50–75	167 (57.0)	73 (52.9)	47 (65.3)	47 (56.6)	
> 75	105 (35.8)	60 (43.5)	24 (33.3)	21 (25.3)	
BMI *(mean* ± *SD)* (kg/m^2^)	23.1 ± 4.0	20.0 ± 1.9	23.9 ± 0.5	27.8 ± 3.8	< 0.001^b^
Gender (%)					
Male (*n*, %)	136 (46.4)	55 (39.9)	35 (48.6)	46 (55.4)	0.073^a^
Female (*n*, %)	157 (53.8)	83 (60.1)	37 (51.4)	37 (44.6)	
Diabetes (%)					
Yes (*n*, %)	240 (81.9)	109 (79.0)	61 (84.7)	70 (84.3)	0.470^a^
No (*n*, %)	53 (18.1)	29 (21.0)	11 (15.3)	13 (15.7)	
Hypertension (%)					
Yes (*n*, %)	125 (42.7)	62 (44.9)	30 (41.7)	33 (39.8)	0.739^a^
No (*n*, %)	168 (57.3)	76 (55.1)	42 (58.3)	50 (60.2)	
LVEF (%) *(mean* ± *SD)*	40.9 ± 16.2	40.7 ± 15.6	40.3 ± 16.0	41.7 ± 17.4	0.859^b^
≤ 40% (*n*, %)	158 (53.9)	74 (53.6)	42 (58.3)	42 (50.6)	0.626^a^
> 40% (*n*, %)	135 (46.1)	64 (46.4)	30 (41.7)	41 (49.4)	
NYHA class HF (%)					
II (*n*, %)	108 (36.9)	49 (35.5)	29 (40.3)	30 (36.1)	0.783^a^
III–IV (*n*, %)	185 (63.1)	89 (64.5)	43 (59.7)	53 (63.9)	
Laboratory results					
NT-proBNP (pg/ml) *median (IQR)*	3776 (1672–8806)	5143 (2112–13654)	3298 (1472–7463)	3177 (1262–5237)	0.0004^c^
HbA1c (%) *median (IQ R)*	7.0 (6.2–8.4)	6.7 (6.0–8.3)	7.3 (6.4–8.3)	7.2 (6.3–8.8)	0.210^c^
eGFR (ml/min/173 m^2^) *median (IQR)*	57.5 (43.9–73.4)	56.7 (43.0–72.2)	56.8 (43.2–70.6)	61.8 (46.1–78.8)	0.343^c^
Cholesterol (mmol/L) *mean* ± *SD*	4.03 ± 1.32	4.01 ± 1.31	4.08 ± 1.39	4.02 ± 1.27	0.937^b^
Triglyceride (mmol/L) *median (IQR)*	1.5 (1.1–2.1)	1.4 (1.0–2.0)	1.6 (1.2–2.2)	1.5 (1.1–2.1)	0.043^c^
HDL cholesterol (mmol/L) *mean* ± *SD*	0.95 ± 0.25	0.97 ± 0.23	0.96 ± 0.27	0.92 ± 0.26	0.412^b^
LDL cholesterol (mmol/L) *median (IQR)*	2.4 (1.9–3.1)	2.3 (1.9–3.1)	2.5 (1.8–3.2)	2.4 (1.9–3.1)	0.690^c^

*BMI* body mass index, *LVEF* left ventricular ejection fraction, *NYHA* New York Heart Association, *HF* heart failure, *GFR* glomerular filtration rate, *HDL* high-density lipoprotein, *LDL* low-density lipoprotein, *SD* standard deviation, *IQR* interquartile range

^a^Chi-square test

^b^ANOVA test

^c^Kruskal‒Wallis test

### NT-proBNP concentration in patient subgroups by BMI categories

Table 2 shows the distribution of NT-proBNP levels according to patient subgroups by BMI strata. The NT-proBNP levels in obese heart failure patients were significantly lower than those in the heart failure patients grouped by gender, diabetes, hypertension, heart failure, and NYHA class (*p* < 0.05) (Table 3). In the overweight BMI category, NT-proBNP levels were significantly higher in females than in males (5850 (2363–9308) vs. 1869 (1104–3797), *p* = 0.0017). The NT-proBNP levels were higher in the heart failure patients with EF ≤ 40% than in the heart failure patients with EF > 40% across the three BMI strata (*p* < 0.05). Similarly, there were higher NT-proBNP levels in the NYHA class III–IV heart failure subgroup than in the NYHA class II heart failure subgroup in the obese and lean BMI categories (*p* < 0.05), except for the overweight BMI category (*p* = 0.156). However, NT-proBNP levels in hypertension and diabetes subgroups were not significantly different in each BMI category (Table 2).

**Table 2:** NT-proBNP levels in patient subgroups by BMI categories

Subgroup	Lean (*n* = 138)	Overweight (*n* = 72)	Obese (*n* = 83)	*p* value
Age group				
< 50	4528 (2036–5416)	2837 (2837–2837)	3310 (470–5402)	0.779^c^
50–75	4645 (2021–9794)	3797 (1610–8603)	2570 (1099–5264)	0.055^c^
> 75	7340 (2534–17018)	1867 (833–4596)	3177 (1414–5139)	0.002^c^
* p* value	0.138^c^	0.195^c^	0.847^c^	
Gender				
Male	4528 (1530–11720)	1869 (1104–3797)	3202 (1707–5122)	0.018^c^
Female	5604 (2712–16785)	5850 (2363–9308)	2805 (1099–5237)	0.009^c^
* p* value	0.068^d^	0.0017^d^	0.912^d^	
Diabetes				
Yes	5338 (2419–15086)	3071 (1540–6801)	3139 (1262–5264)	0.0005^c^
No	4082 (1796–9239)	3589 (1324–12934)	4240 (1726–5122)	0.56^c^
* p* value	0.318^d^	0.678^d^	0.940^d^	
Hypertension				
Yes	4812 (1608–13134)	3693 (1867–10282)	2805 (914–5122)	0.04^c^
No	5352 (2455–15276)	2623 (1324–5850)	3189 (1707–5237)	0.002^c^
* p* value	0.478^d^	0.226^d^	0.497^d^	
LVEF (%)				
≤ 40%	5703 (2712–15727)	4270 (1869–9308)	3591 (2164–7127)	0.039^c^
> 40%	3427 (1640–10757)	1867 (636–3776)	1951 (819–4240)	0.002^c^
*p* value	0.029^d^	0.004^d^	0.003^d^	
NYHA class				
II	3220 (1510–5793)	2218 (1324–5927)	1967 (578–3232)	0.039^c^
III–IV	8212 (2851–16785)	3932 (1610–8214)	4240 (1726–7143)	0.006^c^
*p* value	0.002^d^	0.156^d^	0.001^d^	

*BMI* body mass index, *GFR* glomerular filtration rate, *LVEF* left ventricular ejection fraction, *NYHA* New York Heart Association

^c^Kruskal‒Wallis test

^d^Mann‒Whitney test

**Table 3:** Multivariate linear regression with log-transformed NT-proBNP values

Variables	*β* coefficient (95% CI)	*p* value
BMI	− 0.07 (− 0.10 to − 0.04)	< 0.001
Gender (male)	− 0.38 (− 0.64 to − 0.13)	0.004
eGFR	− 0.01 (− 0.02 to − 0.01)	< 0.001
LVEF (≤ 40%)	0.66 (0.40 to 0.92)	< 0.001
NYHA class (III, IV)	0.39 (0.13 to 0.66)	0.004

*BMI* body mass index, *GFR* glomerular filtration rate, *LVEF* left ventricular ejection fraction, *NYHA* New York Heart Association, *CI* confidence interval. Adjusted for age, gender, eGFR, LVEF, and NYHA class

### Relationship between BMI, covariates, and NT-proBNP levels

**Fig. 1:**
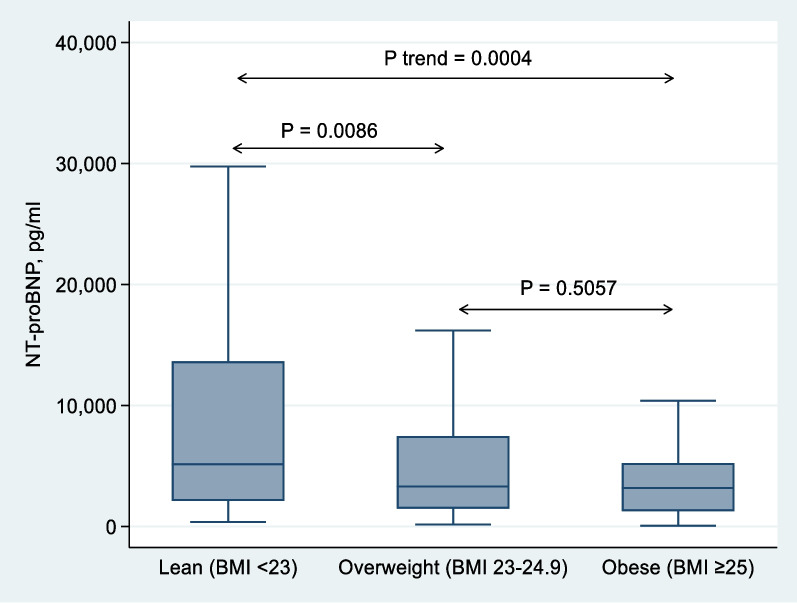
Box plots show median NT-proBNP values in lean, overweight, and obese heart failure patients

**Fig. 2:**
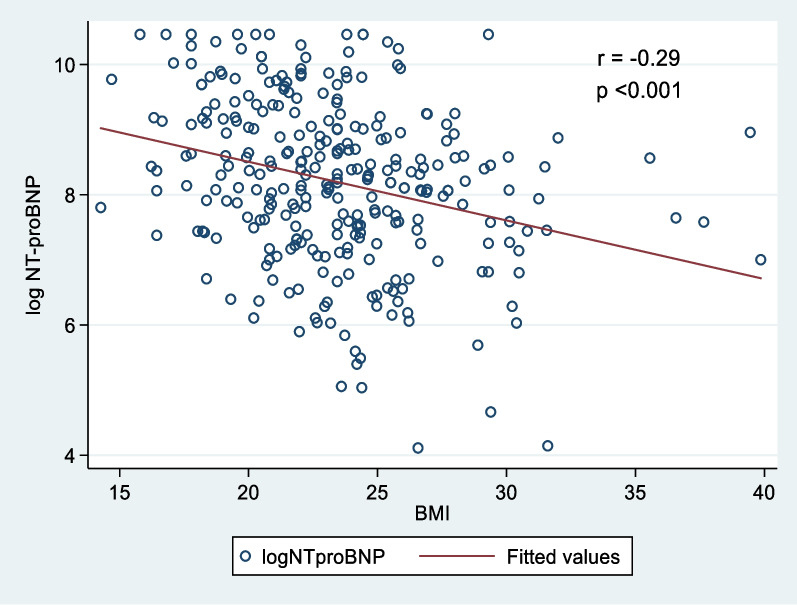
Correlation between log NT-proBNP values with body mass index (BMI)

The median NT-proBNP values ​​in the lean, overweight, and obese patients were 5143 (2112–13,654), 3298 (1472–7463), and 3177 (1262–5237), respectively. The difference in the NT-proBNP levels was statistically significant across all BMI strata (*p* < 0.001). The concentration of NT-proBNP in the obese patients was 38% lower than that in the lean patients (Table 1). The median NT-proBNP was significantly lower in the overweight and obese patients than in the lean patients (*p* < 0.001), whereas the differences in the median NT-proBNP between the overweight and obese patients were not statistically significant (*p* = 0.505) (Fig. 1). There was an inverse association between log NT-proBNP values and BMI (*r* = − 0.29, *p* < 0.001, Spearman correlation) (Fig. 2).

**Fig. 3:**
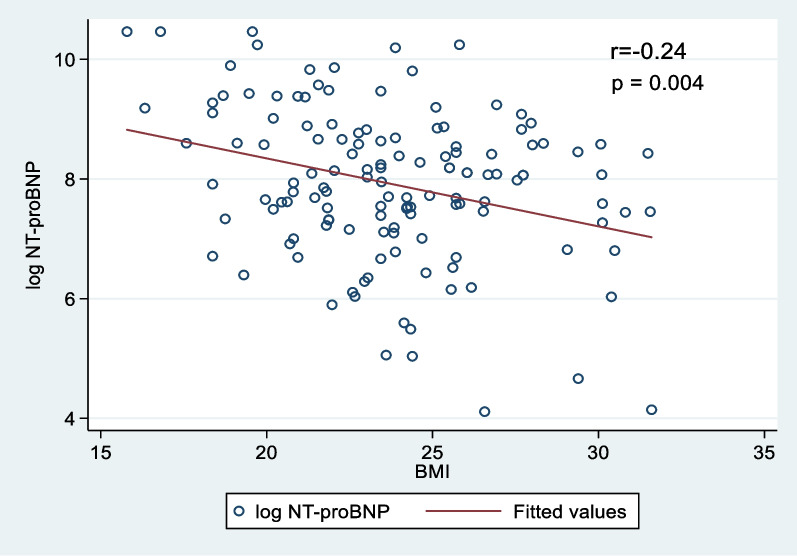
Correlation between log NT-proBNP values with body mass index (BMI) in male

Table 3 demonstrates that BMI was an independent inverse determinant of NT-proBNP values (*p* < 0.001) with multivariable linear regression analysis. Each standard deviation increase in BMI (4 kg/m^2^) was associated with a 7% decrease in NT-proBNP values in the total population because e^(−0.07)^ = 93% (*p* < 0.001), but the decrement in NT-proBNP values was 21% in the male because e^(−0.24)^ = 79% (*p* = 0.004) (Fig. 3). Other independent inverse variables of NT-proBNP values were male gender and eGFR, while LVEF index ≤ 40% and NYHA class III–IV heart failure were significantly directly correlated with NT-proBNP values.

## Discussion

The present study showed an inverse association between BMI and NT-proBNP levels in patients with heart failure. The novelty of this study is that it found other factors independent of or in combination with BMI that affect the NT-proBNP values in heart failure patients and estimated the extent to which BMI changes may affect the NT-proBNP values.

The inverse association between BMI and NT-proBNP levels in heart failure patients found in our study has been reported in many previous studies [1, 11–14, 16–19, 22–26]. The exact mechanism of the inverse association between obesity and NT-proBNP levels remains unclear [2, 23, 27, 28]. It is hypothesized that obesity increases BNP clearance of adipocytes and reduces BNP release from the ventricles [5, 9, 23, 29]. NT-proBNP is a biologically inactive peptide, so it is not cleared by NPR-C (natriuretic peptide clearance receptor-C) of adipocytes, and the decreased release can play an important role in lowering the NT-proBNP levels in obesity [1, 12, 16, 22]. Some studies have shown that NT-proBNP is inversely related to muscle mass rather than fat composition [22, 30].

However, the inverse association between BMI and NT-proBNP values was weak at correlation coefficient *r* = − 0.29 in the present study, although the concentration of NT-proBNP in heart failure patients with obesity was significantly lower (38%) than that in heart failure patients with normal BMI. The weak inverse correlation between BMI and NT-proBNP values was also noted in the previous studies [12, 14, 22, 23, 27], such as Kim HL's study on 1877 heart failure patients who reported an inverse association between BMI and NT-proBNP values with *r* = − 0.232 [17]. Besides, many studies showed that NT-proBNP assay remains valuable in diagnosis and prognosis in both obese and nonobese patients with heart failure, although there is a significant association between NT-proBNP values and BMI [12, 13, 23]. Our study also found that each standard deviation increase in BMI (4 kg/m^2^) was only associated with a 7% decrease in NT-proBNP values. This indicated that the decrease in NT-proBNP levels did not correspond to the increase in BMI. Thus, there will be other independent or combined factors together with BMI affecting NT-proBNP values ​​in heart failure patients.

In this study, the independent variables having a stronger correlation with NT-proBNP levels than BMI were male gender, EF ≤ 40%, and NYHA class III–IV heart failure. Furthermore, diabetes and hypertension may be combined with obesity in affecting NT-proBNP values in heart failure patients to some extent.

Our study found that male gender was an independent inverse determinant of NT-proBNP levels, and the NT-proBNP concentration was significantly lower in male patients than in female patients in the overweight BMI category. In particular, the present study noted that the decrease in NT-proBNP corresponding to each standard deviation increment in BMI was greater in males than in the total population (21% vs. 7%). This finding demonstrated that male gender has a stronger effect on NT-proBNP levels than BMI alone. The fact that the male gender is one of the independent factors inversely related to NT-proBNP levels in heart failure patients has been described in many previous studies [14, 19, 22, 23]. Conversely, Suthahar N. found that the NT-proBNP levels were higher in females than in males, and the author explained the reverse by increased circulating testosterone levels in females with abdominal obesity [25]. The suppressive effect on NT-proBNP levels may be explained by testosterone (male hormone), which tends to inhibit NT-proBNP release [2, 19, 22]. Thus, gender should be considered as a factor in adjustment to NT-proBNP values in obesity.

Heart failure with EF ≤ 40% and NYHA class III–IV heart failure were variables that were linked with an increase in NT-proBNP levels independently of BMI in this study. The NT-proBNP levels in patients with NYHA class III–IV heart failure and heart failure with an EF ≤ 40% were higher than those in patients with NYHA class II heart failure and heart failure with an EF > 40% in both lean and obese patients. These findings were also noted in previous studies [13, 14, 19, 28]. This is explained by severe heart failure leading to increased release of natriuretic peptides, including NT-proBNP, which offsets the NT-proBNP reduction caused by BMI increment [2, 21]. Previous studies have also shown that NT-proBNP levels are a direct predictor of cardiovascular events in both obese and nonobese individuals [1, 11, 12, 19, 24, 31]. It is assumed that NT-proBNP levels are clinically useful in assessing the severity of heart failure irrespective of BMI status.

In the present study, a significant difference was found in NT-proBNP levels in diabetic patients across three BMI categories, while the difference in NT-proBNP levels among the three BMI strata was not significant in patients without diabetes. This finding suggests that diabetes has a certain effect on NT-proBNP values, although diabetes was not found to have a significant association with NT-proBNP levels in the multivariate regression analysis. However, some previous studies have documented that diabetes results in a significant decrease in NT-proBNP levels [11, 19, 23]. This is explained by the increased proBNP glycosylation promoted by elevated blood glucose and insulin resistance status associated with obesity causing a decreased release of NT-proBNP [11, 24].

Theoretically, hypertension exerts a pressure overload on the ventricular myocardium that contributes to NT-proBNP release [2, 21]. However, many studies have shown that hypertension is not significantly associated with NT-proBNP levels [14, 23]. The finding that hypertension was not an independent determinant of NT-proBNP levels was also noted in this study. The reason may be that hypertension patients often have comorbid obesity and/or diabetes, and these disorders reduce the enhancing effect of hypertension on NT-proBNP levels. Indeed, there is an association between obesity and hypertension, and obese individuals have low natriuretic peptide levels that may cause salt retention and excessive adrenergic tone, leading to persistently elevated blood pressure [23].

Thus, diabetes, hypertension, and obesity, which are disorders commonly encountered in heart failure patients, interact with each other to impact the NT-proBNP levels, in which obesity plays a more significant role. Further research is needed to fully explain how these associations affect the NT-proBNP values to manage patients with heart failure more effectively.

All study participants were discharged from the hospital in stable condition. Since this is a cross-sectional study, the analysis of the association between NT-ProBNP and BMI to the outcome of patients with heart failure falls outside the objectives of our investigation. However, numerous studies have reported that the NT-proBNP value is an independent predictor of outcomes in both obese and nonobese patients with heart failure [8, 12, 13, 19, 23, 31]. Further research is also required to confirm this issue in Vietnam.

Because of the inverse relationship between BMI and NT-proBNP values ​​in heart failure patients, some authors recommended BMI-adjusted NT-proBNP values ​​in the diagnosis and prognosis of heart failure [15, 17, 18]. However, this recommendation has not yet been supported by controlled studies, and further clinical trials are needed to verify it [9]. Therefore, the evidence has not been strong enough to change the guidelines for the diagnosis of heart failure. The 2021 ESC guidelines for the diagnosis and treatment of acute and chronic heart failure continue to include NT-proBNP without adjustment based on BMI [6]. The results of this study are consistent with this point of view. The NT-proBNP values remain useful in the diagnosis of heart failure in all BMI categories.

This study has some limitations. Firstly, it was a cross-sectional study, which made it impossible to evaluate the impact of BMI and NT-proBNP values on the prognosis of patients with heart failure. Secondly, the sample size was small compared to previous studies, which may have affected the significance of the results. However, a sample size of 297 cases is still sufficient to yield meaningful results. Furthermore, the study was conducted using rigorous methods, with accurate data collection and appropriate statistical analysis. It was conducted at a sizable 900-bed university hospital over one year, so the results can be considered valid for the study population. To the best of our knowledge, this is one of the first studies in Vietnam to investigate the effect of BMI and other factors on NT-proBNP values in heart failure patients and can provide useful information in the management of heart failure patients.

## Conclusions

Our study found that BMI was inversely related to NT-proBNP values in patients with heart failure. However, the correlation is weak, and the impact of BMI on the NT-proBNP values is small. Furthermore, there are other factors that in combination with or in addition to BMI affect NT-proBNP values ​​in patients with heart failure. These findings show that NT-proBNP values retain their validity in the diagnosis of heart failure irrespective of BMI levels.

## Data Availability

The datasets used and analysed during the current study are available from the corresponding author on reasonable request.

## Abbreviations

BMI: Body mass index
EF: Ejection fraction
ESC: European Society of Cardiology
eGFR: Estimated Glomerular filtration rate
HDL: High-density lipoprotein
HĐĐĐ-ĐHYD: Hội đồng đạo đức—Đại học Y Dược TPHCM
IQR: Interquartile range
LDL: Low-density lipoprotein
LVEF: Left ventricular ejection fraction
NPR-C: Natriuretic peptide clearance receptor-C
NT-proBNP: N-terminal pro-B-type natriuretic peptide
NYHA: New York Heart Association
WHO: World Health Organization
SD: Standard deviation
